# Water Stress, Irrigation and Concentrations of Pentacyclic Triterpenes and Phenols in *Olea europaea* L. cv. Picual Olive Trees

**DOI:** 10.3390/antiox8080294

**Published:** 2019-08-08

**Authors:** Raquel Jiménez-Herrera, Beatriz Pacheco-López, Juan Peragón

**Affiliations:** Biochemistry and Molecular Biology Section, Department of Experimental Biology, Campus Las Lagunillas, University of Jaén, 23071 Jaén, Spain

**Keywords:** *Olea europaea*, maslinic acid, oleanolic acid, erythrodiol, uvaol, betulinic acid, oleuropein, tyrosol, hydroxytyrosol, water stress

## Abstract

Pentacyclic triterpenes and phenols are two types of bioactive molecules found in olive trees that have important activities related to health and disease prevention. Triterpenes, including oleanolic acid, maslinic acid, erythrodiol and uvaol, show antitumoral activities, and phenols such as oleuropein, tyrosol, and hydroxytyrosol are natural antioxidants. The concentration of these metabolites is considered a marker of the quality of olives and olive oil. In recent years, a lack of rain water has caused important economic losses relating to olive trees grown in Jaén, Spain. In this work, we investigated the effect of water stress by drought on the concentration of pentacyclic triterpenes and phenols in the fruits, leaves, stems and roots of cv. Picual olive trees, by comparing the concentration found in water-stressed versus irrigated plants. We used HPLC-UV/Vis and HPLC-MS to identify and determine the concentration of each individual compound. Our results showed that important changes in the concentration of these compounds are produced in response to water stress in different organs. The total content of most of these compounds in the fruits was significantly reduced, affecting their quality and production.

## 1. Introduction

Low water availability is one of the main factors that limits production in the agricultural growing region of the Mediterranean Basin and south of Spain. The low level of rain water in recent years can be considered a climatic change that has begun to affect this region. The overall effect is an important reduction in overall annual precipitation and an increase in the air temperature [1]. In Jaén, the main olive-oil-producing Spanish province, olive trees are mainly rain fed. When water is scarce, plants develop different physiological responses to avoid desiccation, such us regulation of root water uptake and transpiration [2], induction of antioxidant activity and osmotic adjustments [3], and alteration of hormonal content [4]. Several transduction pathways are implicated in the synchronisation of the stress gene response mediated by different transcription factors [5]. Polanco et al. [6] related the resistance capacity of olive trees to gene regulation mediated by different transcription factors that adjust the growth and water uptake capacity of the roots.

Olive trees can survive under prolonged periods of drought through biochemical, morphological and physiological adaptations; nevertheless, water stress by drought is one of the main abiotic stresses that affects fruit growth, and compromises normal fruit tree development. In order to prevent important economic losses in this region, increased irrigation is required. In recent years, many studies have shown how olive trees have adapted to these conditions [1,2,7]. Water availability affects the overall development of the tree and fruit composition, leading to changes in the flavour of olive oil [8,9,10]. The concentrations of free fatty acids, sterols and terpenes change in response to water availability [11,12]. Additionally, the total content of phenolics in irrigated olive trees is lower than in rain fed trees. Nevertheless, no studies exist about the effects of drought on the expression and concentration of pentacyclic triterpenes, or specific phenols such as oleuropein (Ole), tyrosol (Tyr) and hydroxytyrosol (HTyr).

Pentacyclic triterpenes are a group of plant secondary compounds found in relatively high concentrations in olive trees, and have important applications in health and disease prevention. The main ones detected are maslinic acid (2α, 3β)-2,3-dihydroxyolean-12-en-28-oic acid, MA), oleanolic acid (3β-hydroxy-olean-12-en-28-oic acid, OA), erythrodiol (olean-12-ene-3b,28-diol, EO) and uvaol (12-ursen-3-beta,28-diol, UO). They are all molecules made up of 30 carbons grouped in five six-carbon cycles, that have different substitutes. These molecules are synthesised from acetyl-CoA, which is converted into active isoprene. Six molecules of active isoprene condensate to form (3S)-2,3-oxidosqualene, a common precursor for these triterpenes. Oxidosqualene cyclases or triterpene cyclases participate in the synthesis of these compounds [13]. These active biological compounds are present in plants used in traditional medicine and, although more evidence based on human clinical trials is required [14], these compounds show antioxidant [15,16,17], anti-inflammatory [18], anti-microbial [19] and anti-tumour [20,21] activities. The presence and concentration of these compounds in olive oil is considered a quality index of the product [22].

With respect to phenolic compounds, Ole (2-(3,4-dihydroxyphenyl)ethyl [(2S,3E,4S)-3-ethylidene-2-(β-D-glucopyranosyloxy)-5-(methoxycarbonyl)-3,4-dihydro-2H-pyran-4-yl]acetate) is the major constituent of the secoiridoid family in the olive tree, and is present in high concentrations in the leaves [23]. It is responsible for the bitter taste and pungent aroma of olive oil. Ole is a heterosidic ester of elenolic acid and dihydroxyphenylethanol, and can be hydrolysed to hydroxytyrosol, elenolic acid, oleuropein aglycone and glucose [24]. Tyr (4-(2-hydroxyethyl)phenol) and HTyr (4-(2-hydroxyethyl)-1,2-benzenediol) are the most representative phenolics of olive fruits and olive oil. Both occur freely or in the form of esters of secoiridoid elenolic acid. Both compounds share a phenolic substituted at the para position with a hydroxyethyl chain. HTyr has an additional OH group near the other OH group, converting it to one ortho-diphenol [25]. These phenolic compounds have important biological properties related to their antioxidant and anti-inflammatory activities [25,26].

The aim of this study was to investigate the effects of water stress by drought on the concentration of pentacyclic triterpenes and Ole, Tyr, and HTyr in the fruit, leaf, stem and root of cv. Picual olive trees. These are the main organs of the olive tree implicated in the physiological response against water stress; the root is responsive in water absorption, the stem is responsive in water transport, the leaf is the main organ using water for photosynthesis and evapotranspiration, and the fruit is the main annual commercial product of the plant.

## 2. Materials and Methods

### 2.1. Drugs

The analytical or HPLC-grade chemical compounds used as reagents were obtained from Fluka (Buchs, Switzerland), Extrasynthèse (Z.I. Lyon-Nord, Genay, France) and SIGMA (St. Louis, MO, USA).

### 2.2. Experimental Design, Olive Tree Material and Sampling

**Figure 1:**
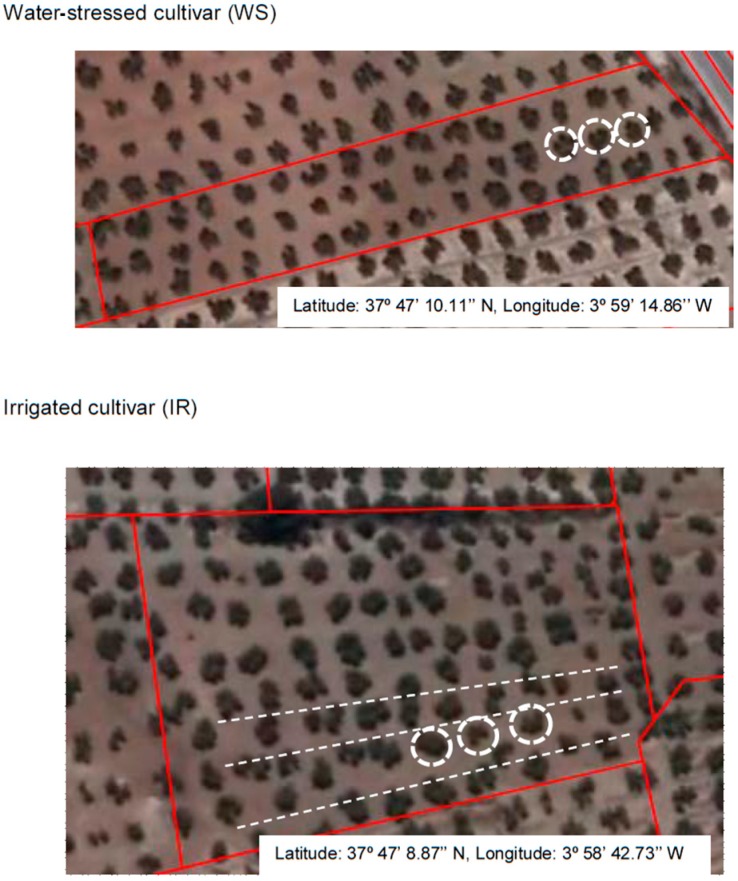
Air photography of cultivars used in this study. The olive trees that were sampled are marked with a white circle. In the lower panel, the positions of the irrigation tubes are marked with a white dotted line. Red lines show the limits of each orchard. The photography was obtained from the Geographical Information System (SIGPAC) of the Ministerio de Agricultura, Pesca y Alimentación, Spain.

**Figure 2:**
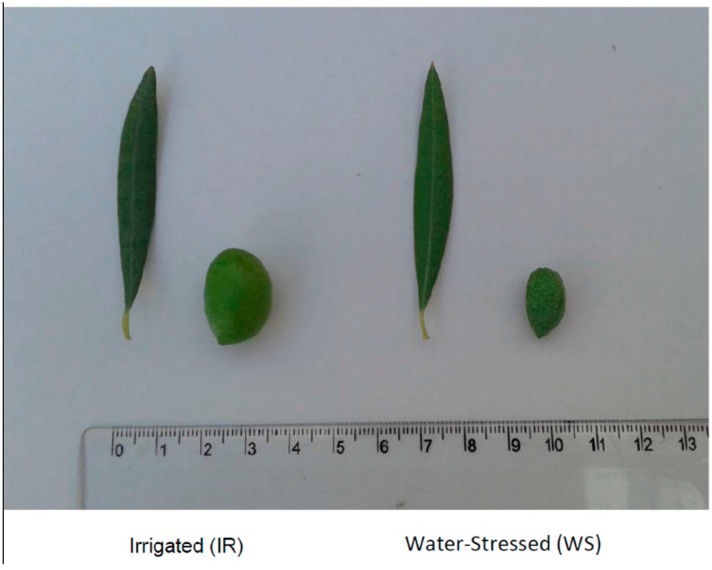
Aspects of fruits and leaves of the irrigated (IR) or water-stressed (WS) olive trees used in this study.

One hundred-year-old *Olea europaea* L. cv. Picual olive trees grown under traditional cultivation methods were used for this study. The sampled trees were located in two different cultivars; one was water-stressed (WS), that only received rain water, and the other was irrigated (IR), located in the Comunidad de Regantes de Torredonjimeno, Jaén, Spain (Figure 1). Both orchards had similar soil conditions and the same climatic and cultivation conditions. The samples were picked on 30 September, 2016. During the four months prior to picking, the rainfall received by the WS trees was 20 L/m^2^, so these trees, fruits and leaves were highly water stressed (Figure 2). The other experimental group, IR trees, had an underground irrigation system with integrated dripping. Tertiary tubes that distributed water were placed 35 cm deep, and were 2 m from each olive trunk. The irrigation caudal was 1.600 L per hectare and hour. During the four months before the samples were picked, the IR olive trees were irrigated one day a week, for 24 h. These trees, fruits and leaves did not experience water stress during the experimental period.

In each orchard, three trees were chosen, and from each orientation in each tree, five 25 cm segments of branches with fruit near the apical end were collected. The leaves and fruit were separated from the branches, and all the leaves and fruit from all three trees were pooled. From the pool of fruit, the ripeness index was determined using a colour evaluation of the skin and flesh [27]. The branches were cut into pieces of 5 cm length. Adventitious roots located near to the surface, with a diameter of 2 mm, were extracted. The different pools of samples were divided into five replicates and frozen in liquid nitrogen (−70 °C) until they were analysed.

### 2.3. Extraction and Quantification of Triterpenic Compounds

We followed the procedure described in Peragón [27] for extraction and analysis of the triterpenic compounds of leaves, fruits, stem and roots of cv. Picual olive trees. At the beginning, the water content of each sample was determined by weighing 3 g of pulverised tissue and drying this in an oven at 55 °C for 2 days, to a constant weight. After cooling, the samples were reweighed.

**Figure 3:**
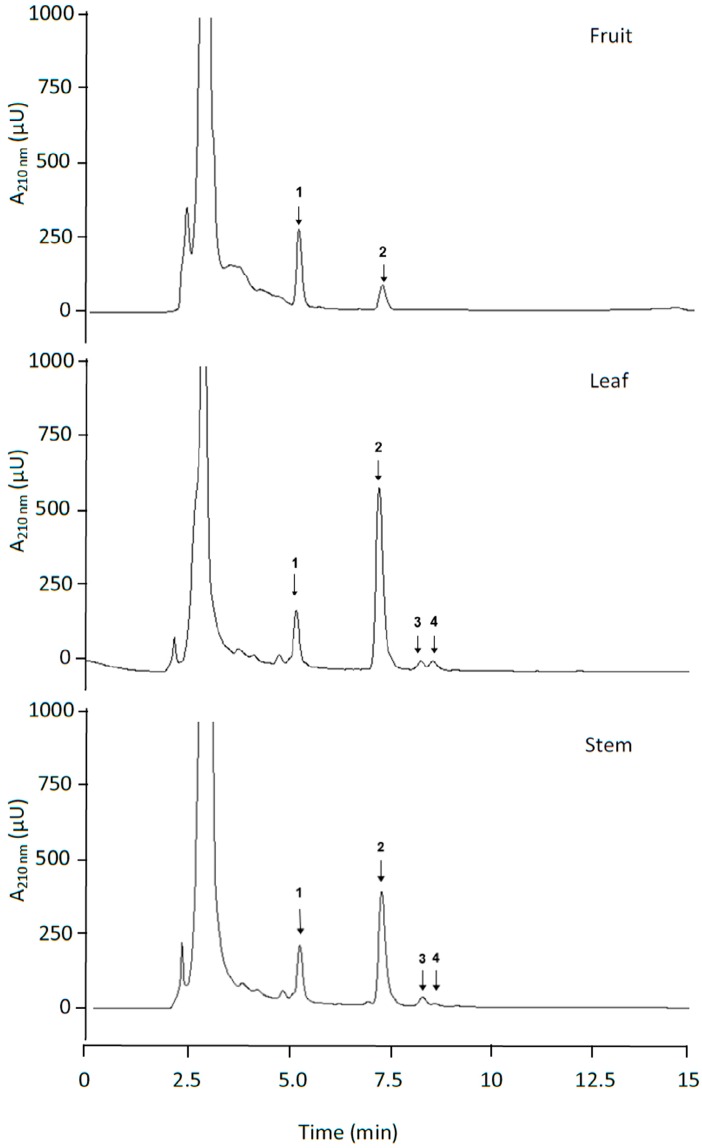
Chromatograms obtained after reverse phase HPLC-UV/Vis analysis of fruit, leaf and stem olive tree triterpenic extracts. Compound 1—maslinic acid, 2—oleanolic acid, 3—erythrodiol, 4—uvaol.

**Figure 4:**
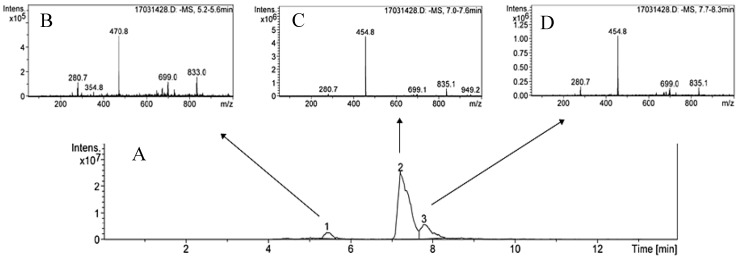
Chromatograms obtained after reverse phase HPLC-UV/vis (Panel (**A**)) and HPLC-MS (Panel (**B**–**D**)) analysis of root olive tree triterpenic extracts. Compound 1—maslinic acid, 2—betulinic acid, 3—oleanolic acid.

For the extraction of the triterpenoids, 0.125 g of dried tissue was mixed with 1.5 mL of methanol:ethanol (1:1, v:v), and was vigorously vortexed for 1 min. The samples were centrifuged at 7700× *g* for 5 min at 4 °C. The supernatants were collected with the residue re-extracted five times with the same volume of methanol:ethanol. All supernatants obtained after five extractions were mixed and evaporated using a Speed-vac. The residue was dissolved with 1 mL of methanol. In this methanolic sample, the analysis of triterpenoids was undertaken using two chromatographic systems: the HPLC-UV/Vis and HPLC-MS/MS systems. Reverse-phase chromatography was applied using a Spherisorb ODS-2 (Waters Corporation, Milford, CT, USA) column (2.5–4.6 mm, 5 µm). A Shimadzu HPLC system including two pumps, a column-heater module, and a UV-Vis detector was used, operating with LC-Solutions software (Shimadzu Corporation, Kyoto, Japan). An isocratic elution was applied using methanol:water with acetic acid (pH = 3.1) (92:8, v:v) for 20 min, with a flow of 0.8 mL min^−1^. The absorbance at 210 nm was recorded during elution. Chromatograms for the leaves, fruits, stem and roots are shown in Figure 3 and Figure 4. The triterpenoids were identified and quantified using the external standard method, as indicated in Peragón [27].

### 2.4. Extraction and Quantification of Phenolic Compounds

We followed the procedure described in Ortega-García et al. [28,29] for the extraction, analysis and quantification of phenolic compounds in the fruits, leaves, stems and roots of olive trees. Samples of different organs were pulverised with liquid nitrogen and homogenised in 80% methanol (1:4, w/v). After shaking in a vortex, the methanolic phase was removed and the residue was re-extracted three times. All the methanolic phases were pooled and washed with hexane two times. Finally, the mixture was centrifuged at 1500× *g* for 5 min. The resulting methanolic phase was used to analyse the phenolic compounds by high-performance liquid chromatography (HPLC), and for the measurement of total phenol content by spectrophotometry.

**Figure 5:**
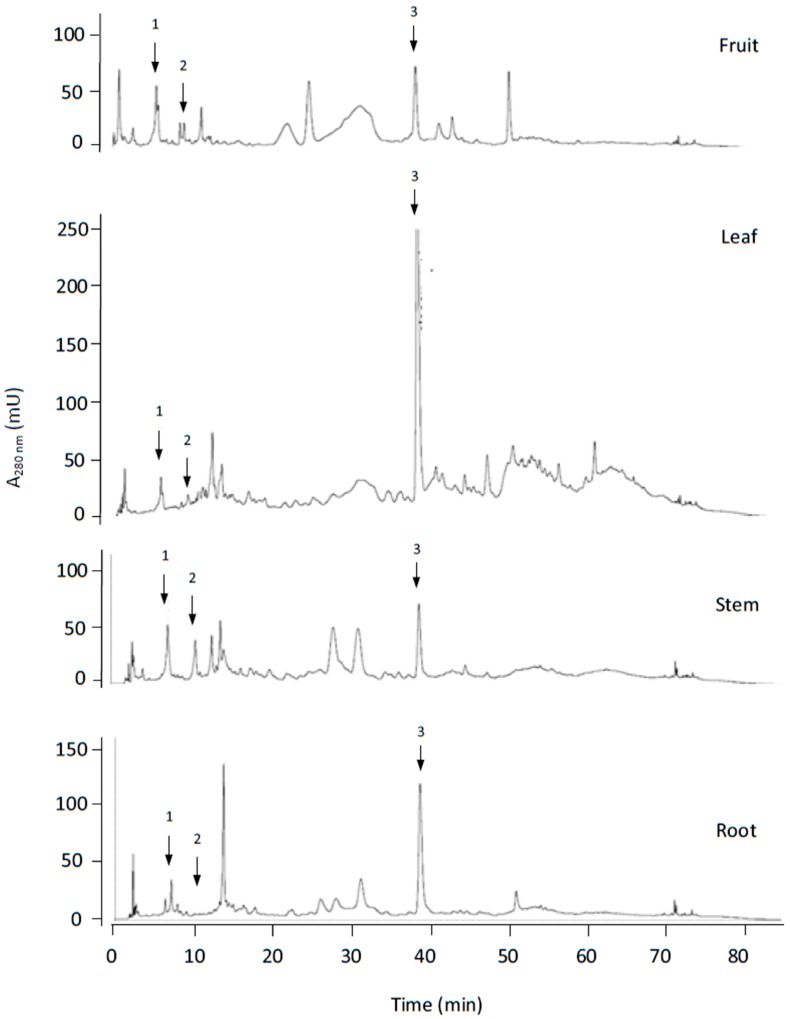
Chromatograms obtained after reverse phase HPLC-UV/Vis analysis of fruit, leaf, stem and root olive tree phenolic extracts. Compound 1: hydroxytyrosol, 2: tyrosol, 3: oleuropein.

HPLC analyses of the methanolic extracts were undertaken using the same HPLC-UV/Vis and HPLC-MS/MS system previously described. We used a reverse-phase Spherisorb ODS-2 column and the Shimadzu HPLC system. The volume of sample injected into the column was 20 μL. The elution gradient used for separation has been previously described in Ortega-García et al. [28,29]. Chromatograms for the leaves, fruits, stem and roots are shown in Figure 5. Ole, HTyr and Tyr were identified and quantified using the external standard method, as indicated in Ortega-García et al. [28,29].

### 2.5. Statistical Analysis

Results are expressed as the mean ± standard error of the mean (S.E.M.). Data were analysed by a one-way analysis of variance. The differences between means were analysed using a Student’s *t*-test. The criterion of significance was taken as *p* < 0.05. Statgraphics Centurion XVI.I Software was used to complete the statistical analysis.

## 3. Results

The present work was conducted on two cv. Picual olive trees under similar culture, fruit charge, and soil conditions in Jaén (southern Spain), differentiated by the supply of water during the period of fruit growth. In Table 1, the weights and humidity of different samples picked from the IR or WS cultivars can be seen. The fruit and pulp weights in the WS samples are 64% and 76% lower than in the IR samples, respectively. This indicated the dramatic effects of water stress on the mass production of olive trees. Differences were lower in stone weight and there were no differences in seed weight. The effect of water stress by drought was clearly evidenced by the lower water concentration of all the organs, with the lowest values found for the roots and leaves, respectively. 

**Figure 6:**
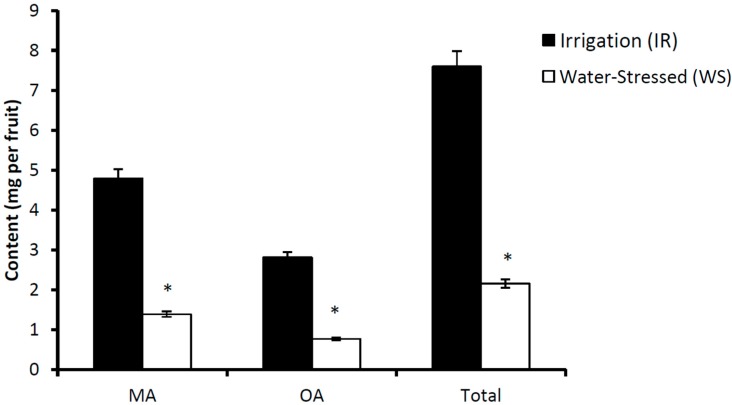
Content of triterpenes (mg) of irrigated (IR) or water-stressed (WS) fruits of cv. Picual olive trees. The content of each triterpene was calculated by multiplying the concentration, expressed as mg per g of fresh weight, by the fruit pulp weight. MA—maslinic acid, OA—oleanolic acid, Total: Sum of maslinic acid and oleanolic acid. For comparisons between the irrigation and water stress conditions, * *p* < 0.05.

The effects of irrigation on the concentration of triterpenoids in the fruits, leaves, stems and roots of cv. Picual olive trees are shown in Table 2. MA and OA are the only two triterpenoids found in the fruits. When comparing the values found in both experimental groups, 12% and 25% lower concentrations of MA and OA are found in IR fruits, respectively. Nevertheless, when the results are expressed in absolute terms of mg per fruit (Figure 6), the total content of MA, OA or total triterpenoids in the IR samples are more than three-fold higher than in the WS samples. Therefore, the total content of both triterpenoids in IR fruits is nearly 8 mg per fruit, and in WS is nearly 2 mg per fruit.

**Figure 7:**
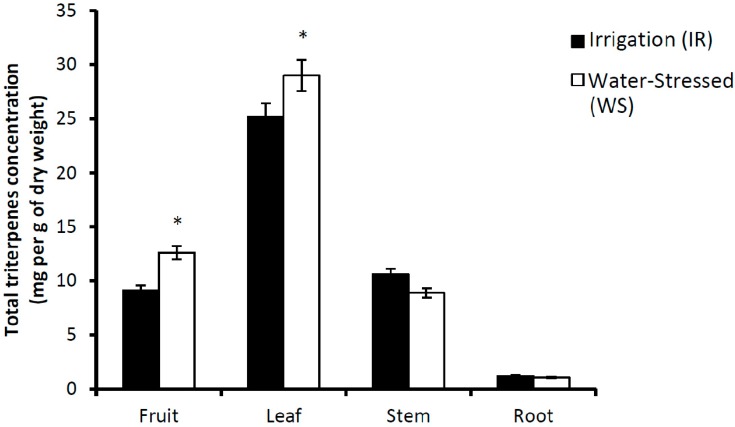
Concentration of total pentacyclic triterpenes found in irrigated (IR) or water-stressed (WS) cv. Picual olive tree samples. For comparisons between the irrigation and water stress conditions, * *p* < 0.05.

In the leaves of the olive trees, MA, OA, EO and UO were detected and quantified. Total concentration of triterpenoids in this organ was calculated as the sum of the individual concentration that is the highest found when compared with the rest of the organs studied (Figure 7). No significant differences in the concentrations of these compounds were found between leaves of the IR or WS samples, with the exception of the concentration of UO, expressed as mg per g of dry weight, which in the WS trees was 179% higher than in the IR condition (Table 3).

In the stems of the olive trees, MA, OA, EO and UO were also detected and quantified. The total concentration was similar to that found in the fruits, and lower than that in the leaves (Figure 7). Irrigation only influenced the EO concentration in this organ. The values found in the WS samples were 51% lower than in the IR samples (Table 2).

We also determined the triterpenoid concentration in the roots of the olive trees. We identified betulinic acid (3β-hydrosy-lup-20(29)-en-28-oic acid, BA) as the main triterpenoid compound found in the roots, together with MA and OA. The sum of the concentrations of the three triterpenic acids in this organ was the lowest found in this study. No differences were found in the concentration of these compounds in response to irrigation.

**Figure 8:**
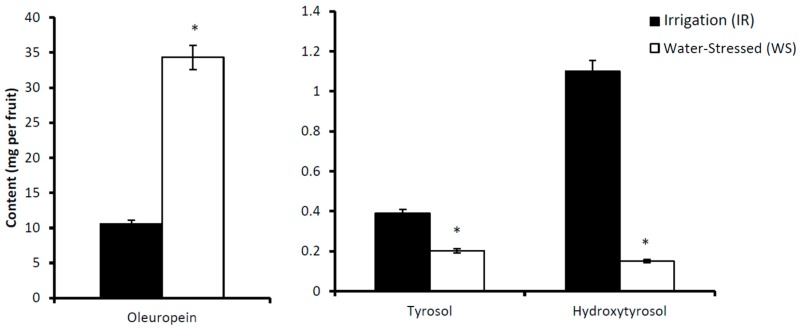
Content of phenolics (mg) of irrigated (IR) or water-stressed (WS) fruits of cv. Picual olive trees. The content of each phenol was calculated by multiplying the concentration, expressed as mg per g of fresh weight, by the fruit pulp weight. For comparisons between the irrigation and water stress conditions, * *p* < 0.05.

In this work we also determined the effect of irrigation on concentration of phenolic compounds such as Ole, Tyr and HTyr in the fruits, leaves, stem and roots of cv. Picual olive trees. The results are shown in Table 3. In the fruits, the concentration of Ole significantly increased by 11.5-fold in response to water stress. This increment lead to the total content of this compound in the WS samples being two-fold higher than in the IR samples (Figure 8). On the other hand, the total content of Tyr and HTyr were significantly lower in the WS samples than in the IR samples. For Ole, a similar behaviour to that described in fruit was found in the stem, while the Tyr concentration decreased (Table 3).

In the leaf and root, a significant decrease in the Ole concentration, expressed as mg/g dry weight, was produced in response to water stress, although no significant change was found when it was expressed as mg/g fresh weight.

## 4. Discussion

The results of the present study showed that an appropriate water supply is needed during the first part of fruit development to obtain the optimal growth of olives in a traditional olive tree cultivar. Water stress by drought during the first part of fruit development produced a dramatic weight loss of the whole fruit, pulp and stone that compromised the normal growth of the olives. These reductions imply important losses in the harvest, likely leading to important economic losses for the farmer. In this situation, in response to water stress by drought, our results showed that although the concentration of MA and OA increased, the total content of MA and OA decreased, resulting in fruits with a low weight and a low content of triterpenes. Moreover, a significant decrease in the content of Tyr and HTyr has also been reported in WS samples. Tyr and HTyr are the main antioxidant phenolic compounds found in olive fruit. This implies that a loss of fruit quality is also produced, which could lead to a loss of quality of the resulting olive oil. An appropriate irrigation water supply is needed to support the weight and quality of olives. Therefore, as found by Masmoudi et al. [30], a reduction in rain water associated with climate change will result in an increase in the irrigation demand to maintain a traditional olive ground. This is the main type of olive ground in the south of Spain and is mainly rain-fed maintained, contrary to the new types of high density and super-high density olive tree orchards that are reliant on irrigation. Therefore, new climatic conditions and the new type of olive tree orchard will increase the need for irrigation water growth significantly in order to maintain optimum growth and optimum fruit composition.

The high concentration and content of Ole found in fruits following water stress by drought is an interesting finding. Ole is the compound that is mainly responsible for the embittered flavour and stability of olive oil. This indicates that the fruit maintained stability in a situation characterised by scarce water availability. 

The changes described here in the fruits are part of the metabolic adaptation to water stress by drought in this species. Other studies have described changes related to osmotic adjustment [31], alteration of transpiration [6,32], the induction of antioxidant activity [3], or the alteration of hormonal content [4]. In our work, in response to drought, the changes described in the fruits are considered the most important changes detected in the four organs after a water stress situation. During the first part of fruit development and after water stress by drought, olive trees try to maintain reproductive growth and oil content accumulation over vegetative growth, suggesting higher sink strength for reproductive growth than for vegetative growth [33].

Moreover, during water stress by drought, important changes have been described in the leaves to adapt to the low water availability. Leaf water deficiency affects the efficacy of photosynthesis, influencing the closing of stomata, the diffusion of CO_2_ into the leaf, and the functionality of photosystem II and rubisco [1]. In our work, we did not find changes in the concentration of pentacyclic triterpenes in the leaves, indicating that the synthesis of OA, MA, EO and UO are maintained even during drought. In the case of UO, the concentration is even is increased. Further, coinciding with these changes, the concentration of HTyr in the leaves significantly decreased in response to drought. 

The stem is the organ that transports water, nutrients and other molecules between the different parts of the plants. The levels of pentacyclic triterpenes and specific phenols determined in this study in the stems were similar to those found in the fruits. In response to water stress by drought, we found a decrease in the EO and Tyr concentrations, while Ole concentration significantly increased.

Furthermore, in this work we show the pentacyclic triterpenoids found in olive tree roots. MA, OA and BA were identified by us. BA was distinguished of OA and ursolic acid—two compounds with the same molecular weight but different chromatographic mobility. BA has been described in many species of plants [34], and has been reported to possess different biological properties, such as anti-inflammatory, anti-cancer and antioxidant functions [35]. In olive trees, BA is only found in the root organ, in which it has a specific function. In response to water stress by drought, no differences between the IR and WS samples were found in this study, indicating that water restriction does not affect the concentration of these compounds in this organ.

In conclusion, in this work we report the changes found in the concentrations and content of MA, OA, BA, EO, UO, Ole, Tyr and HTyr in the fruit, leaf, stem and roots of cv. Picual olive trees cultivated by the traditional method, in response to water stress by drought. These changes are part of the adaptation mechanism of this plant to the climatic conditions in this location, and our findings are evidence for the need for an appropriate availability of water during the development of the fruit in order to obtain optimum growth and quality.

## Figures and Tables

**Table 1:** Ripeness index, fresh weights and humidity of the organs of olive tree growth under irrigation or rain-fed conditions.

Parameter	Irrigation (IR)	Water-Stressed (WS)
Ripeness index	0.50 ± 0.03	0.50 ± 0.02
Fruit weights (g)	2.32 ± 0.13	0.83 ± 0.03 *
Stone weights (g)	0.68 ± 0.03	0.43 ± 0.01 *
Pulp weights (g)	1.64 ± 0.12	0.39 ± 0.03 *
Seed weights (g)	0.05 ± 0.01	0.05 ± 0.01
Humidity of fruits (%)	55.00 ± 3.03	42.42 ± 0.07 *
Humidity of leaves (%)	48.18 ± 0.05	35.05 ± 0.30 *
Humidity of stems (%)	45.56 ± 1.02	37.11 ± 0.04 *
Humidity of roots (%)	42.44 ± 0.89	34.05 ± 1.39 *

The fruit weight was the fresh weight of the whole olive fruit. The stone weight was the fresh weight of the olive fruit without pulp. The pulp weight was obtained as the difference between the fruit weight and stone weight. The seed weight was determined by cutting the stone, extracting the seed and weighing it. Values are means ± standard error of the mean. For comparisons between the irrigation and water stress conditions, values followed by * are significantly different (*p* < 0.05).

**Table 2:** Effects of irrigation on the concentration of triterpenoids in fruits, leaves, stems and roots of cv. Picual olive trees.

Compound	Fruit		Leaf		Stem		Root	
	Irrigation (IR)	Water-Stressed (WS)	Irrigation (IR)	Water-Stressed (WS)	Irrigation (IR)	Water-Stressed (WS)	Irrigation (IR)	Water-Stressed (WS)
Maslinic acid mg/g dry weight	6.00 ± 0.30	8.39 ± 3.53 *	3.28 ± 0.53	2.98 ± 0.22	2.10 ± 0.13	1.82 ± 0.28	0.015 ± 0.004	0.016 ± 0.008
mg/g fresh weight	2.91 ± 0.20	3.53 ± 0.33 *	1.81 ± 0.44	1.36 ± 0.16	1.02 ± 0.08	1.00 ± 0.27	0.008 ± 0.003	0.010 ± 0.006
Oleanolic acid mg/g dry weight	3.13 ± 0.16	4.20 ± 0.30 *	18.92 ± 2.61	21.83 ± 2.31	7.17 ± 0.58	6.30 ± 0.78	0.060 ± 0.021	0.051 ± 0.022
mg/g fresh weight	1.71 ± 0.08	1.98 ± 0.26	10.00 ± 1.36	10.50 ± 2.44	3.38 ± 0.37	2.99 ± 0.93	0.032 ± 0.013	0.031 ± 0.015
Betulinic acid g/g dry weight	nd	nd	nd	nd	nd	nd	1.133 ± 0.382	1.013 ± 0.370
mg/g fresh weight	nd	nd	nd	nd	nd	nd	0.627 ± 0.013	0.604 ± 0.271
Erythrodiol mg/g dry weight	nd	nd	0.92 ± 0.30	1.29 ± 0.21	1.00 ± 0.05	0.47 ± 0.14 *	nd	nd
mg/g fresh weight	nd	nd	0.48 ± 0.14	0.58 ± 0.10	0.47 ± 0.04	0.24 ± 0.12 *	nd	nd
Uvaol mg/g dry weight	nd	nd	2.05 ± 0.48	2.88 ± 0.44	0.31 ± 0.06	0.29 ± 0.07	nd	nd
mg/g fresh weight	nd	nd	0.48 ± 0.11	1.34 ± 0.12 *	0.15 ± 0.03	0.16 ± 0.06	nd	nd

Values are means ± standard error of the mean. In each row and/or each organ, for comparisons between the irrigation and water stress conditions, values followed by * are significantly different (*p* < 0.05).

**Table 3:** Effects of irrigation on the concentration of phenolics in the fruits, leaves, stems and roots of cv. Picual olive trees.

Compound	Fruit		Leaf		Stem		Root	
	Irrigation (IR)	Water-Stressed (WS)	Irrigation (IR)	Water-Stressed (WS)	Irrigation (IR)	Water-Stressed (WS)	Irrigation (IR)	Water-Stressed (WS)
Oleuropein mg/g dry weight	12.27 ± 1.60	153.21 ± 0.51*	62.00 ± 0.63	49.20 ± 3.07 *	8.42 ± 0.01	92.04 ± 0.23 *	62.05 ± 0.31	43.86 ± 0.05 *
mg/g fresh weight	7.04 ± 0.65	85.80 ± 0.29	32.00 ± 0.32	33.48 ± 2.12	4.57 ± 0.01	59.00 ± 0.15 *	36.86 ± 0.19	31.23 ± 0.04
Tyrosol mg/g dry weight	0.62 ± 0.05	0.88 ± 0.02 *	0.18 ± 0.02	0.27 ± 0.05	1.29 ± 0.09	0.78 ± 0.05 *	0.07 ± 0.01	0.14 ± 0.01 *
mg/g fresh weight	0.26 ± 0.02	0.50 ± 0.01 *	0.09 ± 0.01	0.17 ± 0.04	0.72 ± 0.05	0.47 ± 0.05 *	0.04 ± 0.01	0.10 ± 0.01 *
Hydroxytyrosol mg/g dry weight	1.81 ± 0.03	1.17 ± 0.17 *	0.69 ± 0.05	0.39 ± 0.05 *	1.34 ± 0.02	1.39 ± 0.08	0.71 ± 0.12	0.59 ± 0.03
mg/g fresh weight	0.73 ± 0.02	0.44 ± 0.05 *	0.36 ± 0.02	0.24 ± 0.04 *	0.73 ± 0.01	0.89 ± 0.05	0.41 ± 0.06	0.41 ± 0.01

Values are means ± standard error of the mean. In each row and/or each organ, for comparisons between the irrigation and water stress conditions, values followed by * are significantly different (*p* < 0.05).

## Abbreviations

EO	erythrodiol
HPLC-UV/Vis	ultraviolet-visible high-performance liquid chromatography system
HPLC-MS	mass high-performance liquid chromatography system
HTyr	hydroxytyrosol
IR	irrigated
MA	maslinic acid
OA	oleanolic acid
Ole	oleuropein
Tyr	tyrosol
UO	uvaol
WS	water-stressed
